# Distribution and internal correlations of corneal astigmatism in cataract patients

**DOI:** 10.1038/s41598-021-91028-2

**Published:** 2021-06-01

**Authors:** Yuanfeng Jiang, Ying Qin, Shaochong Bu, Hong Zhang, Xiaomin Zhang, Fang Tian

**Affiliations:** Tianjin Key Laboratory of Retinal Functions and Diseases, Tianjin Branch of National Clinical Research Center for Ocular Disease, Eye Institute and School of Optometry, Tianjin Medical University Eye Hospital, Tianjin Medical University, Tianjin, 300384 China

**Keywords:** Diseases, Eye diseases, Refractive errors

## Abstract

The aim of the study is to explore the distribution patterns and internal correlations of the morphological parameters of the cornea in patients with age-related cataract. The Pentacam HR was used to measure anterior corneal astigmatism (ACA), posterior corneal astigmatism (PCA), total corneal astigmatism (TCA) and keratometric corneal astigmatism (KCA). With age, the proportion of with-the-rule (WTR) ACA decreased from 65.31% to 23.63%, while the against-the-rule (ATR) ACA increased from 26.53% to 56.20%. PCA exceeded 0.50 D in 9.14% of eyes, while 76.35% of them were ATR. The magnitude of ACA was positively correlated with PCA in the whole sample, with a more significant correlation in WTR eyes (s_r_ = 0.349, *P* < 0.001). The vector summation effect of PCA to ACA changed from compensation to augmentation with aging. In 57.53% of WTR eyes, KCA was overestimated by an average of 0.21 ± 0.17 D, while it was underestimated by 0.38 ± 0.27 D in 87.62% of ATR eyes. In conclusion, among age-related cataract patients, ACA and TCA gradually shifted from WTR to ATR with aging, while most PCA remained as ATR. Ignoring the age-related changes and real PCA might cause overestimation of WTR astigmatism and underestimation of ATR astigmatism.

## Introduction

The prevalence of corneal astigmatism exceeding 1.0 dioptre (D) has been found to be about 30% among cataract patients^1,2^. At present, toric intraocular lens implantation during cataract surgery is considered to be an effective and a reliable way to correct pre-existing corneal astigmatism^3^. However, the clinical outcomes are derived largely on the accurate estimation of total corneal astigmatism (TCA) preoperatively, which is contributed by both the anterior and posterior corneal surfaces.

Optical biometric methods, such as keratometry and corneal topography, can only acquire information about the anterior corneal surface and derive a simulated keratometric corneal astigmatism (KCA) value using the standard corneal refractive index (1.3375 for most equipment). Based on the Scheimpflug principle, Pentacam HR (Oculus Inc., Wetzlar, Germany) can be used to obtain information on the posterior corneal surface and extract the personalized TCA of an individual using the ray tracing technique^4–6^. Its repeatability and agreement with other systems in evaluating the anterior and posterior corneal surfaces have been assessed in previous studies^7–9^.

To the best of our knowledge, only few reports have assessed large samples of elderly Chinese with corneal astigmatism using Pentacam HR. We used the data obtained via Pentacam HR to evaluate the distribution pattern of corneal astigmatism, correlation between posterior corneal astigmatism (PCA) and anterior corneal astigmatism (ACA), vectoral effect of PCA on ACA, and possible evaluation error of KCA in a Chinese population.

## Methods

### Subjects

The preoperative biometric data of 2013 patients (2013 eyes) diagnosed with age-related cataract who underwent examination with Pentacam HR at the Tianjin Medical University Eye Hospital from October 2016 to October 2018 were analysed. The exclusion criteria were as follows: irregular astigmatism, pterygium, keratoconus, keratoleucoma, history of ocular surgery or trauma, recent history of wearing contact lenses, and any other ocular disorders that could affect the corneal curvature or its image acquisition with Pentacam HR. Personal identifiable information was removed from all cases during the study to protect privacy. The study was approved by the Ethics Committee of Tianjin Medical University Eye Hospital and the requirement for retaining informed consent was waived by the Ethics Commission. All procedures were performed in accordance with the relevant guidelines and regulations.

### Examination

The steep radius (R_s_) and flat radius (R_f_) of the anterior and posterior corneal surfaces along the 15° ring centred on the corneal vertex are the only exactly corresponding dataset that can be directly extracted from the Pentacam database, which were entered into the following formulas to calculate the magnitudes of ACA, PCA, and KCA, respectively:

$${\text{ACA}} = \frac{{{\text{n}}_{{{\text{cornea}}}} - 1}}{{{\text{R}}_{{\text{S}}} \,{\text{of}}\,{\text{anterior}}\,{\text{corneal}}\,{\text{surface}}}} - \frac{{{\text{n}}_{{{\text{cornea}}}} - 1}}{{{\text{R}}_{{\text{f}}} \,{\text{of}}\,{\text{anterior}}\,{\text{corneal}}\,{\text{surface}}}}$$

where, n_cornea_ is the refractive index of the cornea (1.376) and 1 is the refractive index of air.

 

$${\text{PCA}} = \frac{{{\text{n}}_{{{\text{aqueous}}}} - {\text{~n}}_{{{\text{cornea}}}} }}{{{\text{R}}_{{\text{S}}} \,{\text{of}}\,{\text{posterior}}\,{\text{corneal}}\,{\text{surface}}}} - \frac{{{\text{n}}_{{{\text{aqueous}}}} - {\text{~n}}_{{{\text{cornea}}}} }}{{{\text{R}}_{{\text{f}}} \,{\text{of}}\,{\text{posterior}}\,{\text{corneal}}\,{\text{surface}}}}$$

where, n_aqueous_ is the refractive index of aqueous humour (1.336).

$${\text{KCA}} = \frac{{{\text{n}}_{{{\text{KCA}}}} - 1}}{{{\text{R}}_{{\text{S}}} \,{\text{of}}\,{\text{anterior}}\,{\text{corneal}}\,{\text{surface}}}} - \frac{{{\text{n}}_{{{\text{KCA}}}} - 1}}{{{\text{R}}_{{\text{f}}} \,{\text{of}}\,{\text{anterior}}\,{\text{corneal}}\,{\text{surface}}}}$$

where, n_KCA_ is the standardized corneal refractive index (1.3375).

The magnitude of TCA was assessed directly by the ray tracing technique along the 3-mm ring centred on the corneal vertex, which closely matched the 15° ring. The axis of corneal astigmatism was defined as the steep meridian. Each eye was measured 1 to 3 times to achieve the same calibration standard. Only high-quality Pentacam images with 95% or more valid data, automatically marked “OK” in the results, were used for analysis. All measurements were performed by the same skilled technician.

### Data analyses

To assess the axial distribution of corneal astigmatism among different age groups, the subjects were classified into five age groups: 40–49-year, 50–59-year, 60–69-year, 70–79-year, and ≥ 80-year age group. Astigmatism was classified into three types according to the meridian of the maximal convergent power: with-the-rule (WTR, within 60°–90°), against-the-rule (ATR, within 0°–30° or 150°–180°), and oblique (30–60° or 120–150°). All eyes were divided into three groups (WTR eyes, ATR eyes, and oblique eyes) according to the type of astigmatism affecting the anterior corneal surface. The correlation between the magnitudes of ACA and PCA, vector summation effect of PCA on ACA, and consistency and difference between TCA and KCA were analysed in the three groups, respectively.

The vector summation effect of PCA on ACA was assessed by subtracting the magnitude of ACA from TCA. A positive value indicated that PCA yielded a vector augmentative effect on ACA, while a negative value indicated a compensative effect.

The normality of the data was first assessed using the Kolmogorov–Smirnov test. The proportions of eyes with WTR, ATR, and oblique astigmatism among the different age groups were compared using the chi-square test. Spearman’s correlation analysis and linear regression were used to investigate the relationship between the magnitude of ACA and PCA. The difference between the magnitude of TCA and KCA was compared using the Wilcoxon signed-rank test. The agreement between the TCA and KCA was assessed using the Bland–Altman method with 95% limits of agreement (LoAs) (mean difference ± 1.96 standard deviation). Statistical analyses were performed using SPSS version 20.0 (IBM Corp., Armonk, NY, USA). Diagrams were drawn using SigmaPlot version 14.0 (Systat Software Inc., San Jose, CA, USA) and Astigmatism Double Angle Plot Tool version 110 provided on the online website (ascrs.org). Analysis items with *P* values less than 0.05 were considered statistically significant.

## Results

The present study enrolled 2013 eyes of 2013 patients (719 men and 1294 women) with a mean age of 69.80 ± 9.48 years (range 40–95 years). Table 1 shows the relationship between the magnitude or the type of corneal astigmatism and gender. The correlations between the magnitude of corneal astigmatism and other ocular parameters of the study population, including white-to-white (WTW), central corneal thickness (CCT), anterior chamber depth (ACD), and axial length (AL), are shown in Table 2. ACA and TCA were negatively correlated with WTW, while PCA was positively correlated. ACA was positively correlated with AL, while PCA and TCA were not significantly correlated. Both CCT and ACD had no significant correlation with the magnitude of ACA, PCA, or TCA.

**Table 1:** Comparison of the corneal astigmatism magnitude and type between male and female patients.

Parameters	Magnitude of astigmatism (D)			Proportion of astigmatism types
	ACA	PCA	TCA	WTR/ATR/oblique
Male	0.99 ± 0.71	0.27 ± 0.15	1.06 ± 0.75	36.02%/45.06%/18.92%
Female	1.07 ± 0.77	0.27 ± 0.15	1.15 ± 0.79	31.84%/47.99%/20.17%
z/χ^2^	− 2.223	− 0.639	− 3.204	3.639
*P* value	0.026	0.523	0.001	0.162

D, dioptre; ACA, anterior corneal astigmatism; PCA, posterior corneal astigmatism; TCA, total corneal astigmatism; WTR, with-the-rule; ATR, against-the-rule.

**Table 2:** Correlation between corneal astigmatism and other ocular parameters.

Parameters	WTW (mm) (n = 1469)	CCT (mm) (n = 2013)	ACD (mm) (n = 2013)	AL (mm) (n = 1641)
Mean value	11.29 ± 0.45	542.3 ± 31.34	3.10 ± 0.47	23.82 ± 1.81
**Correlations with**				
ACA	s_r_ = − 0.111 *P* < 0.001	s_r_ = 0.017 *P* = 0.437	s_r_ = − 0.006 *P* = 0.771	s_r_ = 0.071 *P* = 0.004
PCA	s_r_ = 0.093 *P* < 0.001	s_r_ = 0.029 *P* = 0.193	s_r_ = 0.043 *P* = 0.052	s_r_ = 0.049 *P* = 0.046
TCA	s_r_ = − 0.158 *P* < 0.001	s_r_ = 0.031 *P* = 0.169	s_r_ = − 0.036 *P* = 0.107	s_r_ = 0.037 *P* = 0.136

ACA, anterior corneal astigmatism; PCA, posterior corneal astigmatism; TCA, total corneal astigmatism; WTW, white-to-white; CCT, central corneal thickness; ACD, anterior chamber depth; AL, axial length.

Table 3 shows the mean magnitudes and cumulative percentages of corneal astigmatism. PCA was 0.50 D or higher in 184 eyes (9.14%). KCA and TCA exceeded 1.00 D in 736 eyes (36.56%) and 999 eyes (49.63%), respectively. According to the type of ACA, 671 (33.33%), 945 (46.95%), and 397 (19.72%) eyes were WTR, ATR, and oblique, respectively. However, the PCA in 1537 eyes (76.35%) was of the ATR type.

**Table 3:** Distribution of the magnitude of corneal astigmatism.

Parameters	Magnitude (D)		Cumulative percentage (%)			
	Mean ± SD	Range	≤ 0.25 D	≤ 0.50 D	≤ 1.00 D	≤ 2.00 D
ACA	1.04 ± 0.75	0–5.44	8.10	23.15	58.62	89.97
KCA	0.94 ± 0.68	0–4.88	9.44	27.12	63.54	92.60
PCA	0.28 ± 0.17	0–1.80	49.33	90.86	99.71	100.00
TCA	1.12 ± 0.78	0–6.50	5.96	24.19	56.14	89.92

SD, standard deviation; D, dioptre; ACA, anterior corneal astigmatism; KCA, keratometric corneal astigmatism; PCA, posterior corneal astigmatism; TCA, total corneal astigmatism.

**Figure 1:**
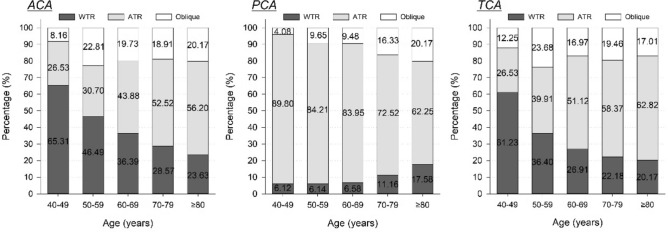
The distribution of astigmatism types in ACA, PCA, and TCA in the different age groups. ACA, anterior corneal astigmatism; PCA, posterior corneal astigmatism; TCA, total corneal astigmatism; WTR, with-the-rule; ATR, against-the-rule.

Figure 1 shows the axial distribution of corneal astigmatism in all age groups. With age, the rate of WTR in ACA decreased from 65.31% (40–49-year) to 23.63% (≥ 80-year), whereas ATR astigmatism increased from 26.53% (40–49-year) to 56.20% (≥ 80-year) (χ^2^ = 77.830, *P* < 0.001). The rate of ATR in PCA decreased slightly from 89.80% (40–49-years) to 62.25% (≥ 80-year) (χ^2^ = 77.355, *P* < 0.001), whereas the trend of WTR posterior astigmatism was unclear. The age trend of TCA was similar to that of ACA (χ^2^ = 69.663, *P* < 0.001). There was no significant difference in gender ratio (male/female) amongst the 40–49-year (22/27), 50–59-year (97/131), 60–69-year (240/441), 70–79-year (234/474), and ≥ 80-year (126/221) age group (χ^2^ = 8.739, *P* = 0.068).

**Figure 2:**
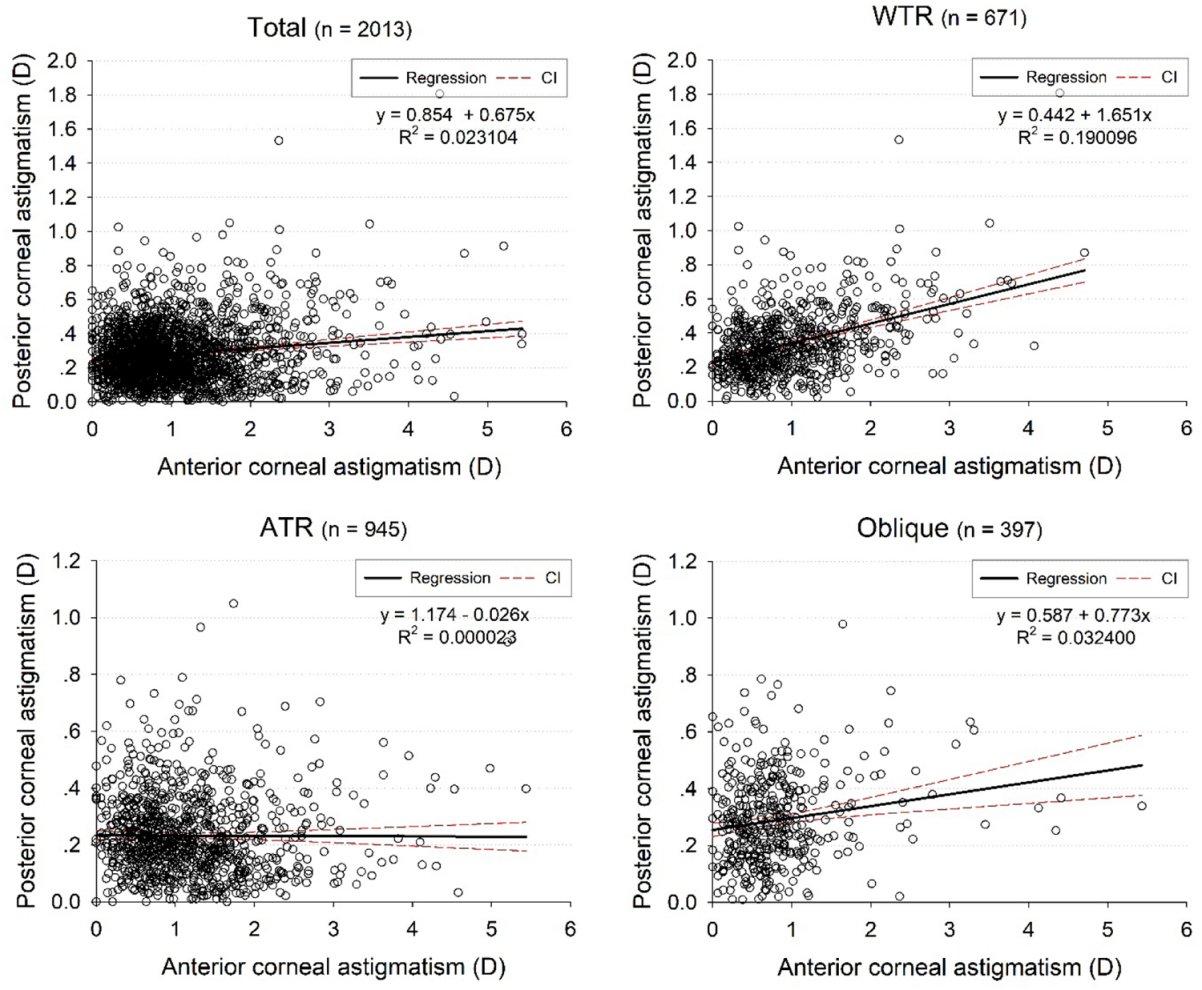
Scatter plots and linear regression of the correlation between the magnitudes of PCA and ACA. D, dioptre; ACA, anterior corneal astigmatism; PCA, posterior corneal astigmatism; WTR, with-the-rule; ATR, against-the-rule.

Scatter plots and regression lines (Fig. 2) illustrated the correlation between PCA and ACA in the entire sample and among the WTR, ATR, and oblique eyes, respectively. Spearman’s correlation analysis showed positive correlations between PCA and ACA in WTR eyes (s_r_ = 0.3490, *P* < 0.001), oblique eyes (s_r_ = 0.1560, *P* = 0.002), and total eyes (s_r_ = 0.0545, *P* = 0.015). However, a negative correlation was found in ATR eyes (s_r_ = − 0.1100, *P* < 0.001).

**Figure 3:**
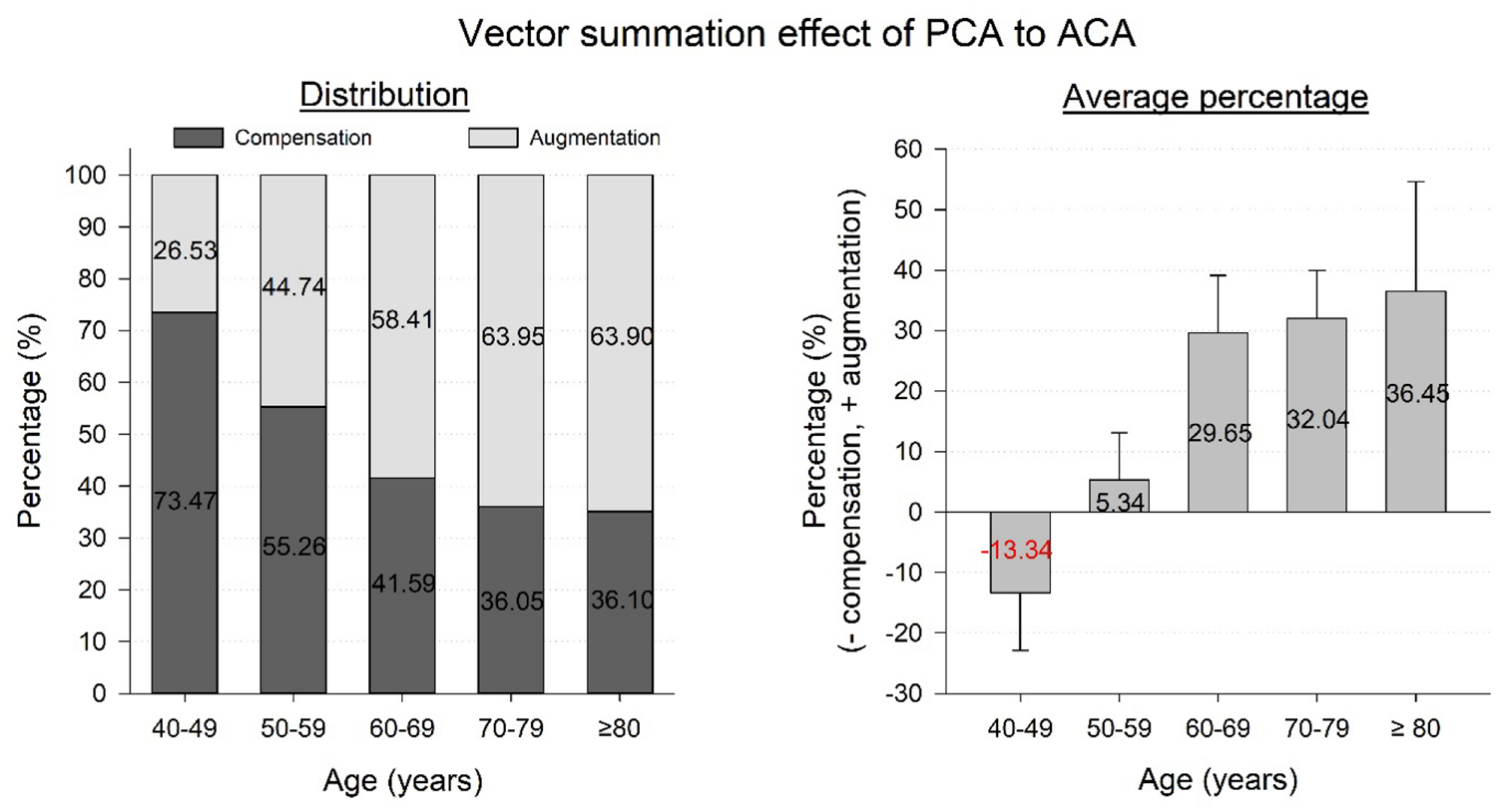
Distribution (left) and average percentage (right) of the vector summation effect (compensation or augmentation) of PCA on ACA in different age groups. ACA, anterior corneal astigmatism; PCA, posterior corneal astigmatism.

In the entire sample, the vector summation effect of PCA on ACA reduced the magnitude of ACA by an average of 0.24 ± 0.21 D (25.39 ± 20.48%) in 832 eyes (41.33%) and increased it by 0.30 ± 0.26 D (65.37 ± 132.59%) in 1181 eyes (58.67%). The proportion of eyes that showed a compensative effect progressively decreased from 73.47% (40–49-year) to 36.10% (≥ 80-year), while the proportion of eyes that showed an augmentative effect increased from 26.53% (40–49-year) to 63.90% (≥ 80-year) (Fig. 3, Left). There was a significant difference in the distribution of the two opposite effects in the different age groups (χ^2^ = 52.998, *P* < 0.001). The mean proportion changed gradually from − 13.34% for compensation in the 40–49-year age group to 36.45% for augmentation in the ≥ 80-year age group (s_r_ = 0.129, *P* < 0.001) (Fig. 3, Right). The turning point occurred in the 50–59-year age group.

**Figure 4:**
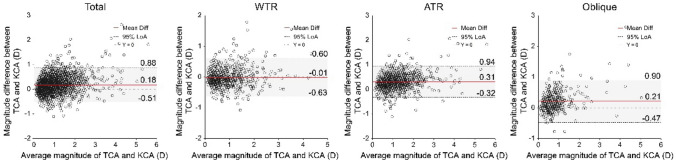
Bland–Altman plots comparing the TCA magnitude to the KCA magnitude. Mean differences are represented by red lines and 95% LoAs are represented by dotted lines. D, dioptre; LOA, limit of agreement; TCA, total corneal astigmatism; KCA, keratometric corneal astigmatism; WTR, with-the-rule; ATR, against-the-rule.

**Figure 5:**
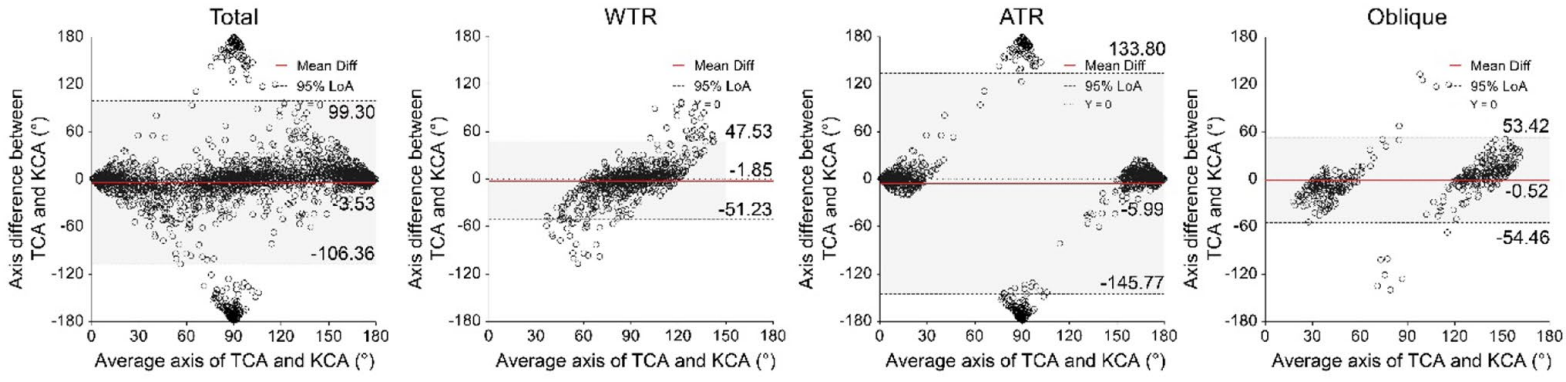
Bland–Altman plots comparing the TCA axes to the KCA axes. Mean differences are represented by red lines and 95% LoAs are represented by dotted lines. °, degree; LOA, limit of agreement; TCA, total corneal astigmatism; KCA, keratometric corneal astigmatism; WTR, with-the-rule; ATR, against-the-rule.

Bland–Altman plots comparing the magnitudes and axes of TCA to KCA are presented in Figs. 4 and 5, respectively. The mean differences (arithmetic estimation errors) in the magnitude of TCA and KCA were 0.18 D, − 0.01 D, 0.31 D, and 0.21 D in the entire sample, WTR, ATR, and oblique eyes, respectively (Fig. 4). In total, 355 (17.64%), 62 (9.24%), 224 (23.70%), and 69 (17.38%) eyes had an absolute error greater than 0.50 D in the entire sample, WTR, ATR, and oblique eyes, respectively. The Wilcoxon signed-rank test showed statistically significant differences between the magnitudes of TCA and KCA in the entire sample, ATR, and oblique eyes (z = 8.358, 9.868, and 5.959, respectively; all *P* < 0.001), but not in the WTR eyes (*t* = − 1.107, *P* = 0.268). The magnitude of TCA was on average 0.21 ± 0.17 D lower in 57.53% WTR eyes than the magnitude of KCA, while it was 0.38 ± 0.27 D higher in 87.62% of ATR eyes and 0.34 ± 0.31 D higher in 74.31% of oblique eyes.

The mean differences between the axes of TCA and KCA were − 3.53°, − 1.85°, − 5.99°, and − 0.52° in the entire sample, WTR, ATR, and oblique eyes, respectively (Fig. 5). In total, 843 (41.88%), 299 (44.56%), 326 (34.50%), and 218 (54.91%) eyes had absolute errors greater than 10° in the entire sample, WTR, ATR, and oblique eyes, respectively. Statistically significant differences between the TCA and KCA axes were demonstrated in the entire sample and ATR eyes (z = − 3.019 and − 2.581; *P* = 0.003 and 0.010), but not in the WTR and oblique eyes (z = − 1.899 and − 0.375, *P* = 0.058 and 0.708).

A total of 1032 eyes (51.27%) had either a KCA magnitude that differed by > 0.50 D from the TCA magnitude or a KCA axis that differed by > 10° from the TCA axis.

**Figure 6:**
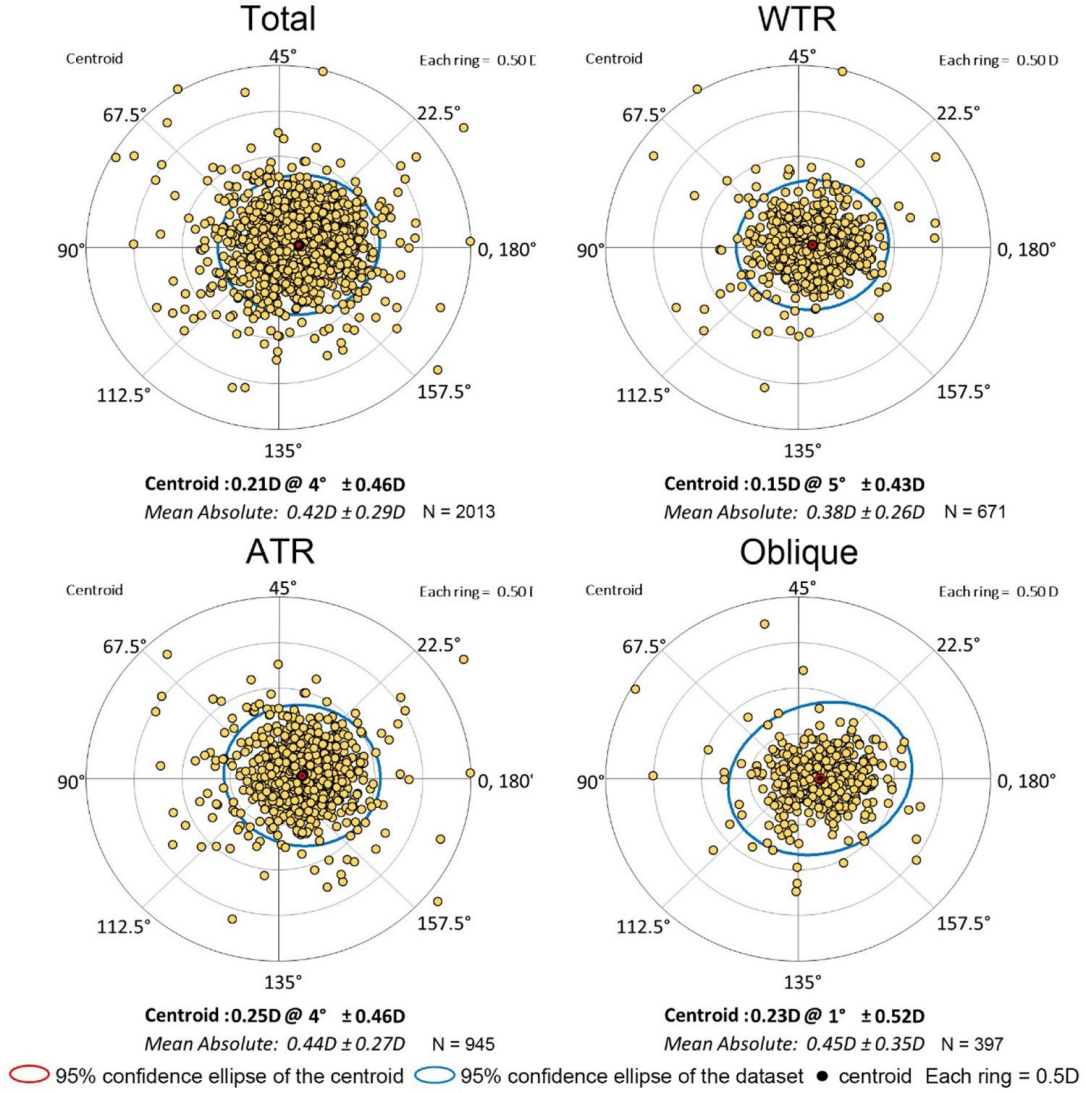
Double-angle plots of difference vectors between TCA and KCA. °, degree; D, dioptre; TCA, total corneal astigmatism; KCA, keratometric corneal astigmatism; WTR, with-the-rule; ATR, against-the-rule; The double-angle plots were created using the Astigmatism Double Angle Plot Tool version 110 provided on the online website (ascrs.org).

The centroids and the distribution of difference vectors between TCA and KCA are shown in Fig. 6 using double-angle plots. In total, 652 (32.41%), 173 (25.77%), 347 (36.68%) and 133 (33.55%) eyes had absolute differences greater than 0.50 D in the entire sample, WTR, ATR, and oblique eyes, respectively.

## Discussion

The precise evaluation of corneal astigmatism is closely related to uncorrected visual quality after cataract surgery, particularly among patients with premium intraocular lens implantation. Moreover, recent evidence indicates the unneglectable role of PCA in astigmatism assessment. Here, we provided a relatively comprehensive perspective on the distribution pattern of corneal astigmatism in a large sample of age-related cataract patients in China and analysed the potential internal relationship among its components.

In the present study, the mean magnitude of TCA was 1.12 D in the population older than 40 years, of which 49.63% exceeded 1.00 D. It is noteworthy that both of the above results were lower in the KCA, which were 0.94 D and 36.56%, respectively. Similarly, the proportions of TCA and KCA over 1.00 D were 40.0%^10^ and 36.04%^1^ in large samples consisting of European adults. It is known that the essential difference between the TCA obtained by Pentacam and KCA is that the former considers the real PCA of each individual, while the latter does not. Our results showed that the average value of PCA was close to 0.3 D, nearly one tenth of which exceeded 0.50 D. Consistent with our findings, prior studies using Pentacam HR have reported mean values of PCA ranging from 0.25 to 0.33 D (Table 4)^10–14^. Collectively, this indicates that PCA is not negligibly small and foreshadows the difference between TCA and KCA. However, regardless of the influence of PCA, at least one third of eyes have clinically significant levels of corneal astigmatism, which need to be corrected during cataract surgery.

**Table 4:** Published magnitudes of mean corneal astigmatism derived from Pentacam HR.

Study^a^	Population	Eyes /patients, n	Mean age, range, (Y)	Mean ± SD, range, (D)				(Percentage, %)
				ACA	PCA	TCA	KCA	PCA > 0.5 D
Tonn ^10^	German	3818/2233	47.5 ± 15.0 20 to 79	1.28 ± 1.01 0 to 8.10	− 0.33 ± 0.18 0 to − 1.35	1.13 ± 0.89 0 to 7.2	1.15 ± 0.90 0 to 7.27	15
Shao ^11^	Chinese	3769/3769	38.17 ± 20.22 18 to 92	_	− 0.33 ± 0.16 − 1.19 to 0.00	_	_	14.27
Naeserk ^12^	Danes	710/710	51.5 ± 18.0 20 to 88	_	0.25, _	_	_	_
Jiang ^13^	Chinese	1976/1976	61.82 ± 13.67 3 to 100	0.92 ± 0.65 0 to 4.5	0.28 ± 0.16 0 to 1.0	1.04 ± 0.68 0 to 4.7	_	_
Miyake ^14^	Japanese	608/608	55.3 ± 20.2 15 to 96	1.14 ± 0.76 0 to 4.9	0.37 ± 0.19 0 to 1.20	_	_	_
Current	Chinese	2345/2013	69.80 ± 9.48 40 to 95	1.05 ± 0.76 0 to 5.76	0.28 ± 0.17 0 to 1.8	1.12 ± 0.78 0 to 6.50	0.94 ± 0.68 0 to 5.20	9.25

Y, years; SD, standard deviation; D, dioptre; ACA, anterior corneal astigmatism; PCA, posterior corneal astigmatism; TCA, total corneal astigmatism; KCA, keratometric corneal astigmatism.

^a^First author.

With regard to axial distribution, both ACA (46.95%) and PCA (76.35%) were mainly of the ATR form among age-related cataract patients, which also explains why the average magnitude of TCA was greater than KCA and corresponds to the phenomenon of rapid shift from the compensative effect of PCA on ACA to augmentative effect after the age of 50 years. In addition, the proportion of ATR eyes classified by TCA (55.44%) was higher than that classified by ACA, suggesting that it might be underestimated if the evaluation is simply based on the anterior surface. However, an investigation of 1976 patients with congenital and age-related cataract conducted in eastern China found that WTR astigmatism was predominant in ACA (43.1%), which might be attributed to the enrolment of younger patients^13^.

In addition, our results revealed the axial distribution patterns of corneal astigmatism with age. With age, the proportion of WTR astigmatism decreased gradually in ACA, being replaced by more ATR types, which was consistent with the results of Ho et al.^15^ However, the dominant position of ATR astigmatism in PCA slightly decreased with age. A similar pattern was previously reported by Shao et al.^11^ and Jiang et al.^13^, while this trend was not obvious in the study conducted by Koch et al.^16^ This inconsistency might be related to the differences in sample size and ethnicity^11,13,16^.

The combination of drift of ACA toward ATR with age and the relative invariability of PCA resulted in the weakening of the compensative effect of PCA on ACA. This soon turned into an increasingly augmentative effect in the elderly beginning at 50–59-year age group. From another perspective, it also explained the higher ATR ratio in TCA than in KCA. Shao et al.^11^ reported that the drift of TCA towards ATR occurred at a rate of 0.45 D per 10 years between the ages of 36 and 68 years. Although the mechanism has not been clarified, most studies have suggested that the change in eyelid anatomy and its decreasing mechanical tension with age may play an important role^17–19^. Other age-related eye features may also contribute to the shift of astigmatism axis, such as the axial length, anterior chamber depth and horizontal corneal diameter, especially in the Asian population^20,21^.

From the results of linear regression and correlation analysis, it could be concluded that there was a positive correlation between the magnitudes of PCA and ACA in the entire sample. However, other researchers have reported discrepancy in the results regarding different types of astigmatism, which could be partly due to the different statistical interpretations of regression lines and correlation coefficients^13,14,16,22,23^. Nevertheless, all previous studies have confirmed the strongest correlation in WTR eyes, which was consistent with our study (s_r_ = 0.3490). Knowledge of these rules prompts us to be aware of the influence of PCA in eyes, particularly in WTR eyes with higher corneal astigmatism in clinical practice.

The Bland–Altman analysis showed wide 95% LoAs for the magnitude and axis between TCA and KCA. In the entire sample, half of the eyes had a magnitude difference exceeding 0.50 D or an axial difference exceeding 10°. Vector analysis showed the values of centroid difference and average difference were about 0.2 D and 0.4 D, of which one third exceeded 0.50 D, especially among ATR eyes. These findings provide supporting evidence for the important role of PCA in the generation of estimation errors and indicate limited interchangeability between TCA and KCA in clinical use. This difference may be attributed to the KCA estimation model, which assumes that the curvature of the anterior and posterior corneal surfaces is fixed in the population and that the meridian of the posterior surface with maximal convergent power intersects that of the anterior surface vertically^24^. Among the WTR eyes, although most PCA (not all) was found to be of the ATR form, the actual augmentative ratio of PCA on ACA may be higher than the pre-set constant in KCA estimation^25^. This assumption might explain why 57.53% of KCA showed an overestimation of 0.21 D on average. For ATR eyes, the steep meridian of PCA was assumed to be horizontal in KCA estimation. In contrast, most (76.35%) of them were actually vertical and produced an ATR refraction power that yielded an augmentative effect on ACA. Therefore, it is not difficult to explain that although the average magnitude of actual PCA in ATR eyes was the smallest, it played the opposite role of the hypothetical PCA. Thus, a mean underestimated value of 0.38 D was found in 87.62% of ATR eyes, 25.97% of which exceeded 0.50 D.

Interestingly, the ACA and TCA of among female patients seemed to be slightly higher than that of male patients, but the difference was not clinically significant. However, there were no statistical differences among the proportions of different astigmatism types between male and female patients. Among other ocular parameters, WTW was negatively correlated with the magnitude of ACA and TCA, and positively correlated with PCA. AL was positively correlated with the magnitude of ACA. This might indicate that a smaller cornea or a longer axis is more likely to exhibit a higher astigmatism. Similar patterns were confirmed by a recent study in 39,986 Chinese cataractous eyes^26^. Further study should be performed to clarify the relationships between corneal astigmatism and other ocular characteristics.

Unfortunately, the ray tracing TCA along the 15° ring, which should have completely corresponded to the measuring range of KCA, ACA, and PCA, could not be extracted directly from the Pentacam database. To some extent, this natural limitation might inevitably cause objective errors in the accuracy of parameter comparison and analysis. The relatively small sample size of 40–49-year age group, due to the onset age characteristics of age-related cataract, may result in some bias to relevant statistical analyses. Other limitations include possible measurement deviations caused by head position, eyelid compression, and the dynamic change of tear film and slight eye movement during the process of image acquisition.

In summary, our results provide a panoramic view of the distribution and internal relationship of corneal astigmatism among patients with age-related cataract. Up to a third of this population has clinically significant astigmatism requiring correction. After the age of 50 years, overall astigmatism of the cornea gradually drifted to ATR with age. Most notably, compelling pieces of evidence indicated that the important role of PCA must not be ignored in corneal astigmatism evaluation. Our study provides epidemiological support for clinical astigmatism correction and paves the way for future work aiming at improving the accuracy of refractive cataract surgery planning.
